# Durability Performance and Corrosion Mechanism of New Basalt Fiber Concrete under Organic Water Environment

**DOI:** 10.3390/ma16010452

**Published:** 2023-01-03

**Authors:** Jun Wei, Zhenshan Wang, Weidong Sun, Runan Yang

**Affiliations:** 1 School of Civil Engineering, Suzhou University of Science and Technology, Suzhou 215009, China; 2 School of Civil Engineering and Architecture, Xi’an University of Technology, Xi’an 710048, China

**Keywords:** basalt textile reinforced concrete, durability, performance degradation, corrosion mechanism

## Abstract

Under corrosive environments, concrete material properties can deteriorate significantly, which can seriously affect structural safety. Therefore, it has important engineering applications to improve the durability performance at a lower economic cost. This paper proposes a new, highly durable concrete using inexpensive construction materials such as basalt fiber, sodium methyl silicate, and inorganic aluminum salt waterproofing agent. With the massive application of sewage treatment projects, the problem of concrete durability degradation is becoming more and more serious. In this paper, five types of concrete are developed for the sewage environment, and the apparent morphology and fine structure of the specimens after corrosion in sewage were analyzed. The density, water absorption, and compressive strength were measured to investigate the deterioration pattern of concrete properties. It was found that ordinary concrete was subject to significant corrosion, generating large deposits of algae on the surface and accompanied by sanding. The new concrete showed superior corrosion resistance compared to conventional concrete. Among other factors, the inorganic aluminum salt waterproofing agent effect was the most prominent. The study found that the strength of ordinary concrete decreased by about 15% in the test environment, while the new concrete had a slight increase. Comprehensive evaluation showed that the combination of basalt fiber and inorganic aluminum salt waterproofing agent had the best effect. Its use is recommended.

## 1. Introduction

Concrete structures serving in harsh environments give rise to huge annual losses due to insufficient durability. With the development of the modern city, the demand for water treatment structure is strong. As a typical corrosive environment, sewage has caused serious corrosion to structures such as sewage tanks and pipelines, greatly shortening their service life and increasing maintenance costs, resulting in immeasurable economic losses. Therefore, it is urgent to improve the durability of concrete in sewage environment.

Domestic and foreign research has been conducted for a long time to explore the corrosion mechanism of sewage on concrete, both physicochemical corrosion [1,2], and microbial corrosion [3,4,5]. Concrete corrosion is mainly affected by microbial redox generation of H_2_S and biological sulfuric acid corrosion [6,7]. Scholars have carried out in-depth studies for H_2_S, biological sulfuric acid corrosion in sewage. Li [8], Jiang [9] et al. studied the effects of H_2_S and relative humidity on concrete sewer corrosion. The results show that the corrosion rates in the gas phase and partially submerged concrete specimens are positively correlated with the concentration of H_2_S, and high relative humidity affects the specimens in the gas phase. Sun et al. [10,11] found that a high concentration of H_2_S impairs the oxidation of sulfide in the pipe, making the concrete surface sulfide absorption decrease after the first increase. Min et al. [12] studied the effect of sulfuric acid pH on concrete corrosion, and the results showed that sulfuric acid corrosion of concrete was most severe when the pH was 2.5. Mahmoodian et al. [13] studied the effect of sulfuric acid concentration and temperature. They found that: the more acidic the solution, the more serious the corrosion, but the change in solution temperature has almost no effect on corrosion. Yuan [14], Xiao [15,16] et al. showed that sulfuric acid would react with cement hydrate to produce gypsum. With the increase of corrosion days, the surface becomes rough, the peeling-off phenomenon occurs, and the elastic modulus, peak strain, and peak stress of concrete are gradually reduced. In short, whether it is H_2_S cavitation or sulfuric acid corrosion, the essence is acid corrosion; with an increase in corrosion time, there is a more pronounced decline in the strength of the concrete. The large amount of H_2_S produced by microorganisms in the sewage is the main reason for the degradation of concrete material properties.

Much research has been carried out by scholars on how to improve the corrosion resistance of concrete materials; the coating method and the addition of highly durable materials are hot research directions. For concrete in a sewage environment, a surface coating of bacterial inhibitor will reduce the bacterial activity [17]; the bacterial repair coating can extend the service life of the structure, which becomes 1.5 times longer than that of ordinary mortar [18]. In adding high-durability materials, the basalt fiber role is more significant and has attracted widespread attention, and many scholars have done a great deal of research on basalt fiber concrete. Basalt fibers have excellent durability with a slow decline in mechanical properties in acid and salt environments [19,20]. Adding basalt fibers to concrete creates a certain tensile effect within it. It improves the mechanical properties, while the denseness of the material and its resistance to penetration are significantly improved, which enhances its corrosion resistance [21,22,23]. However, the fiber content will affect the performance. Too little will weaken the anti-permeability effect; excessive mixing will affect the compactness of the concrete and reduce durability [24,25,26].

Zhou et al. [27] conducted compression, tension, and bending tests on concrete with different basalt fiber contents. It was shown that the mechanical properties of concrete were significantly improved when the volume fraction of basalt fibers was 0.3% and 0.4%; however, the performance of basalt fibers was weakened as the optimum volume fraction was exceeded. Alaskar [28], Lu [29] and Wang [30] et al. indicated that the addition of basalt fibers under high-temperature conditions could resist heat transfer and maintain the stiffness and toughness of concrete, as well as inhibit the carbonation of concrete and improve its chloride penetration resistance and compressive strength. Deng [31] and Li [32] et al. studied modified basalt fibers, finding increased splitting tensile and flexural strength of concrete. Dvorkin et al. [33] found that using the best ratio of steel and basalt fiber reinforcement can avoid segregation within the concrete mixture and improve its uniformity, with steel and basalt fiber reinforced concrete having higher strength. Zhao et al. [34] and Kwak et al. [35] studied the performance of basalt fiber concrete after freeze-thaw cycles. The results showed that incorporating fibers could alleviate the freeze-thaw damage process to concrete and improve fracture performance. However, the bond strength will gradually decrease with the increase of freeze-thaw cycles. In summary, adding basalt fiber has a positive effect on concrete durability performance; the result is pronounced and has good engineering application value.

In this paper, we add various materials, such as water-soluble sodium silicate and inorganic water repellent, to basalt fiber to further enhance the performance of concrete against penetration. This forms a certain protective film inside the concrete to block the corrosion by harmful substances. This paper develops a new type of modified concrete by adding fiber and an anti-seepage agent. It has high mechanical properties and corrosion resistance, and excellent protective effect. This method is novel, practical, simple to operate, low cost, and has good application prospects. This paper describes a corrosion study carried out for this new concrete material, using an organic aqueous solution instead of sewage. The study measured the apparent change pattern after corrosion. The density, water absorption, and compressive strength of the material were also determined. At the same time, microscopic corrosion structure and composition analyses were performed. The results of this paper can provide a basis for the engineering application of this new concrete material.

## 2. Research

### 2.1. Materials and Scheme

Cement: Qinling P · O42.5 ordinary Portland cement; aggregate: coarse aggregate has the particle size of 5 mm~16 mm continuous graded gravel; the fine aggregate is medium sand, and the fineness modulus is 2.6~2.8. Water reducer: polycarboxylic acid high-performance water reducer. Other materials: mineral powder, silica fume, titanium dioxide, alum, quartz sand, sodium methyl silicate, inorganic aluminum salt waterproofing agent, basalt fiber (fiber volume content 0.10%). The test block size is 100 mm × 100 mm × 100 mm; the specific material ratio is shown in Table 1.

The test block was immersed in artificially configured high-concentration organic water, and the high-concentration artificial sewage mix ratio is shown in Table 2. In the test, a batch of test blocks were taken out every 60 d for macro and micro index tests. In the future, advanced machines and network technologies can be used to monitor the test process, scientifically analyze the data, and finally compare it with the test results to get the most rigorous conclusions [36,37].

### 2.2. Corrosion Tests

**Figure 1:**
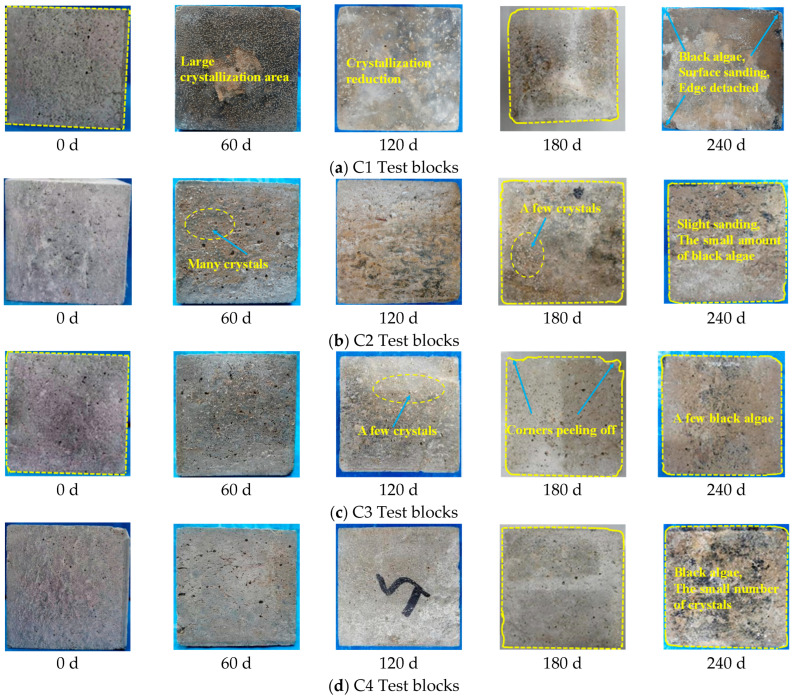

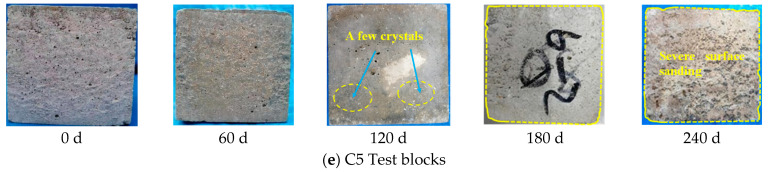
Concrete corrosion phenotypes.

The test will be of five different ratio test blocks immersed in organic water solution. Every 60 d, part of the test blocks is taken out, to observe the corrosion of the test blocks. The changes in the phenological phenomena of concrete with increasing corrosion cycles are shown in Figure 1. The surface darkening (algae deposition) of concrete of each ratio is crystallized. Comparing the appearance of concrete with different ratios at the same age, it is found that C1 and C2 have more crystallization on the surface, and the amount of crystallization decreases with the increase of corrosion time. A small amount of crystallization was found on the surface of C3–C5 concrete, with the least amount of crystallization in C4. By 240 d, the apparent damage of C1 was more severe, and the edges were peeling off. The C2 surface was lightly sanded, C3 and C4 were intact and less affected by corrosion, C5 surface was severely sanded and had powder peeling off. By comparing the apparent corrosion phenomenon, we found that adding basalt fiber, mineral admixture, and water repellent can effectively stop the concrete from being eroded and improve the durability performance of the material.

### 2.3. Strength Tests

Compressive strength can reflect to a certain extent the concrete test block bearing capacity. The strength test was carried out on the test blocks by taking test blocks out every 60 d to observe the effect of corrosion solution on the bearing capacity of concrete with different ratios. The compressive strength test is based on the “Standard for Mechanical Properties Test Methods of General Concrete”, and three test blocks are taken from each group for compressive strength (taking the average value). The testing machine (Shanghai Hualong Testing Instruments Co., Shanghai, China)with displacement control was loaded at a rate of 0.45 mm/min and stopped loading when the specimens reached 75% of the maximum load-bearing capacity.

**Figure 2:**
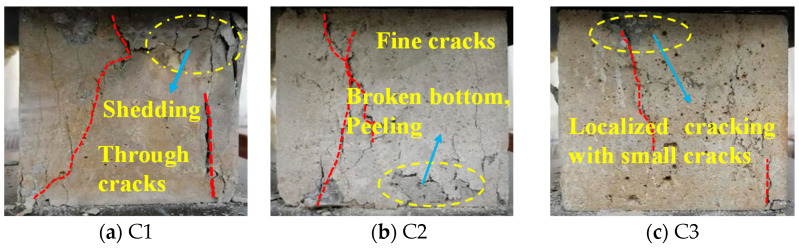

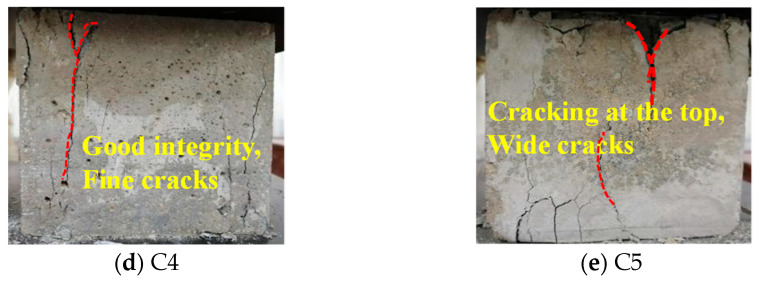
Compressed concrete damage.

The mechanical test of the compressive strength of the concrete after corrosion was carried out. It can be seen from Figure 2 that ordinary concrete C1 has penetrating cracks at the edge, the top is crushed, and a small amount of concrete falls off. Compared with C1, the integrity of the test blocks of C2, C3, and C5 is obviously improved after the compressive test, but the surface has different degrees of damage, mainly fine cracks. C4 has the best overall appearance, with only minor cracks. In the process of compressive tests of concrete, the time from crack to failure of ordinary concrete is very short, due to brittle failure. Comparing the failure process and phenomenon of concrete test blocks with the same age and different proportions horizontally, it can be found that adding basalt fiber can improve the failure form and integrity of concrete after failure and improve the compressive strength of concrete after corrosion. Through the damage of concrete in the strength test, it is found that C4 has the best compressive performance, followed by C3 and C5, which laterally indicates that after adding inorganic aluminum salts, the corrosion resistance of concrete is improved and the compressive strength is enhanced, which is consistent with the conclusions obtained from the corrosion test.

## 3. Methods

### 3.1. SEM Electron Microscopy Scan

**Figure 3:**
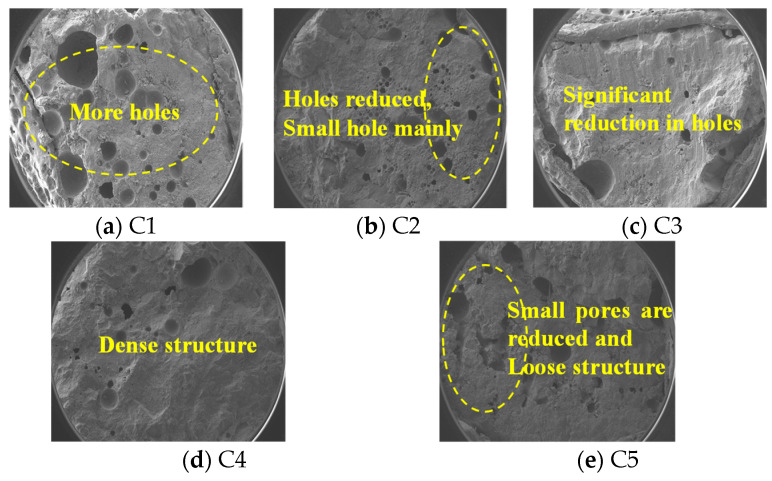
Concrete pore structure changes after corrosion.

Concrete pore structure changes after corrosion. Figure 3 shows that after eight months of corrosion of the concrete surface cement stone, the structure has become loose and porous. C1 has more pores, mainly large pores. C2 has medium and small pores, but the number is less than for C1. It can be seen that the basalt fiber and mineral admixture can slow down the corrosion of the concrete and improve corrosion resistance. C3, C4 cement stone structure does not change much over the period; it is still relatively dense. C4 denseness is the best, indicating that a single admixture of sodium methyl silicate and inorganic aluminum salt waterproofing agent can effectively improve the corrosion resistance of concrete. C5 shows a small, medium and large pores fragmented distribution, and a slightly loose structure, indicating that double admixture of sodium methyl silicate and inorganic aluminum salt waterproofing agent on the corrosion resistance of concrete has a weakening effect. The addition of mineral admixture has a significant impact on reducing the structural porosity and improving the compactness. C1 is ordinary concrete with significant internal defects and more gaps. In addition, after the corrosion of the organic aqueous solution, harmful ions infiltrate through the cracks and react with the cement hydration products, causing some of the cementitious materials to fall off and increasing the pores of C1. This is accompanied by a large number of harmful substances entering the concrete and causing internal corrosion, further increasing the porosity and reducing the compactness, so the phenomenon of C1 damage during corrosion is evident. The strength test under the C1 compressive capacity is also the weakest; in contrast, the C4 structure is dense and less porous, so the apparent damage phenomenon is lighter, and the compressive strength is the highest.

**Figure 4:**
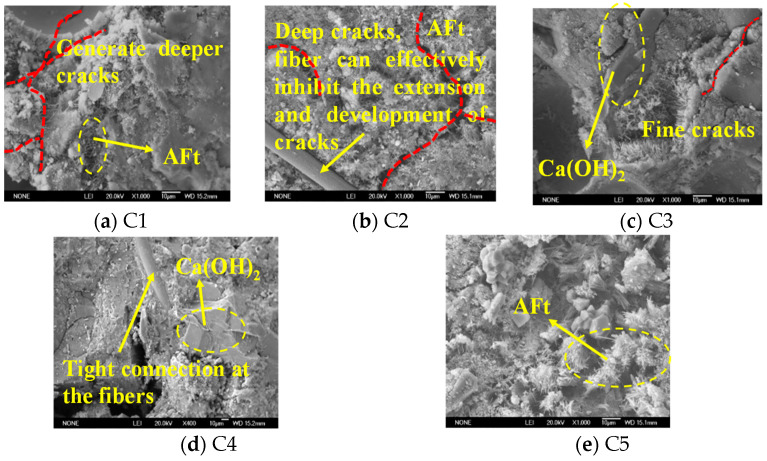
Microscopic morphology of concrete after corrosion.

The SEM microstructure of the concrete after 240 d of corrosion by organic aqueous solution is shown in Figure 4. C1 and C2 were loose on the surface, generating a large number of needle-like ettringite crystals, and almost no Ca(OH)_2_ crystals were observed; both generated deep cracks, but the basalt fibers in C2 inhibited the development of cracks. Hexagonal plate-like Ca(OH)_2_ crystals can be observed in C3 and C4 test blocks, and their internal structures are relatively dense. It shows that adding sodium methyl silicate and inorganic aluminum salt waterproofing agents can effectively improve the corrosion resistance of concrete in a sewage environment. Fibers can also be observed crossing in C4, which are tightly connected to the structure. However, C5, double-doped with sodium methyl silicate and inorganic aluminum salt water repellent, has a slightly loose structure and ettringite formation. The production of calcium alumina in C1 and C2 indicates the intrusion of SO_4_^2−^ ions, causng a reaction with hydration products, while a large amount of Ca(OH)_2_ in C3 and C4 means that sodium methyl silicate and inorganic aluminum salt water repellent can both promote cement hydration and block the infiltration of harmful substances, ensuring that the corrosion resistance of the material is improved.

### 3.2. X-ray Diffraction

**Figure 5:**
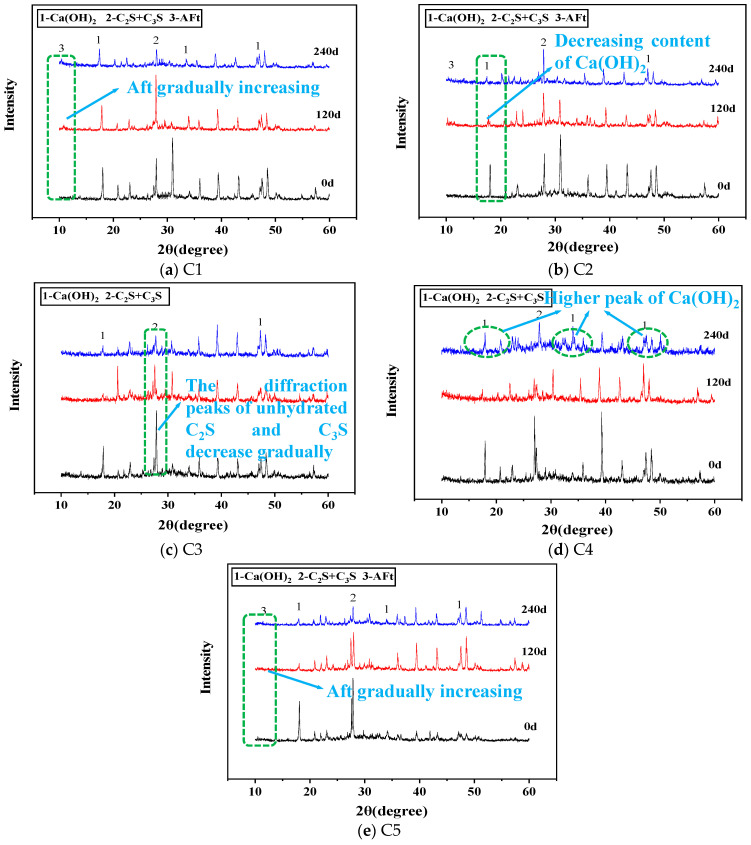
X-ray diffraction of hydration products of concrete with different fitting ratios.

From Figure 5, it can be seen that the diffraction peak of ettringite in C1 is higher than that of all the other matching concrete. SO_4_^2−^ reacts with cement hydration products to form ettringite. Ettringite will produce expansion stress and damage the internal structure of concrete, which is also the main reason for concrete damage. Therefore, the compressive strength of C1 is the lowest. A small amount of ettringite was detected in C2, and the content of Ca(OH)_2_ decreased with the increase of corrosion, but it was still the main component. The diffraction peaks of C_2_S and C_3_S in C3 and C4 decreased significantly; the diffraction peak of Ca(OH)_2_ was very high, and ettringite was not detected. This shows that incorporating sodium methyl silicate and inorganic aluminum salt waterproofing agent is beneficial to cement hydration and effectively prevents the entry of SO_4_^2−^. The diffraction peaks of C_2_S and C_3_S in C5 get lower and lower. It can be seen that the double mixing of sodium methyl silicate and inorganic aluminum salt waterproofing agent has a more apparent promoting effect on cement hydration. Still, a small amount of ettringite is produced. It is possible that the double-doped waterproofing agent has a weakened effect on their respective barrier effects and does not achieve the barrier effect of 1 + 1 > 2. The generation of ettringite will have a certain impact on the strength of concrete, which is consistent with the conclusion of the compressive strength values obtained.

## 4. Results

### 4.1. Density and Water Absorption

**Figure 6:**
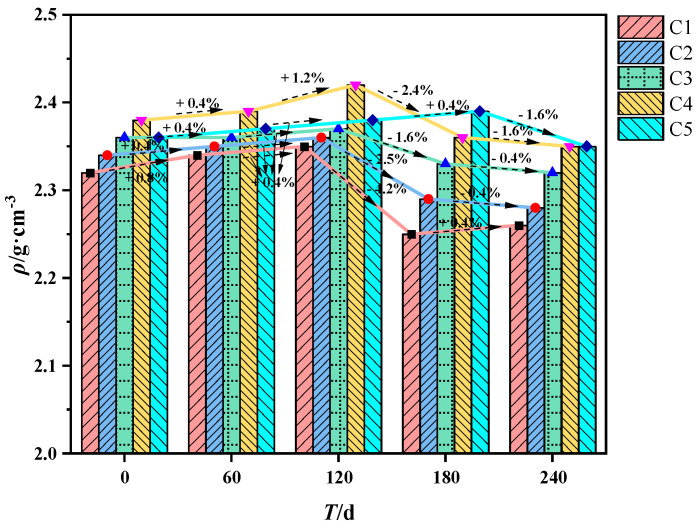
Concrete density variation.

Density and water absorption tests were conducted for each concrete mixing ratio after corrosion, and the results were as follows. The concrete density variation is shown in Figure 6. Overall, the concrete density shows a trend of increasing and then decreasing. It indicates that the crevices in the internal structure first reduce and then grow. The reason is that in the early stage of corrosion, the salt ions in the organic aqueous solution enter the concrete through the gaps. The precipitated crystals fill the internal pores, making the structure dense. Since the accumulation of microorganisms in organic aqueous solution is not enough, the mass loss caused by the corrosion of concrete by the biological acid it produces is not enough to offset the mass of the crystals precipitated in the pores, so the density of concrete increases first. By the late stage of corrosion, with the accumulation of biological acids in the solution, the corrosion of the concrete structure increases, and the mass loss increases significantly, so the density decreases.

**Figure 7:**
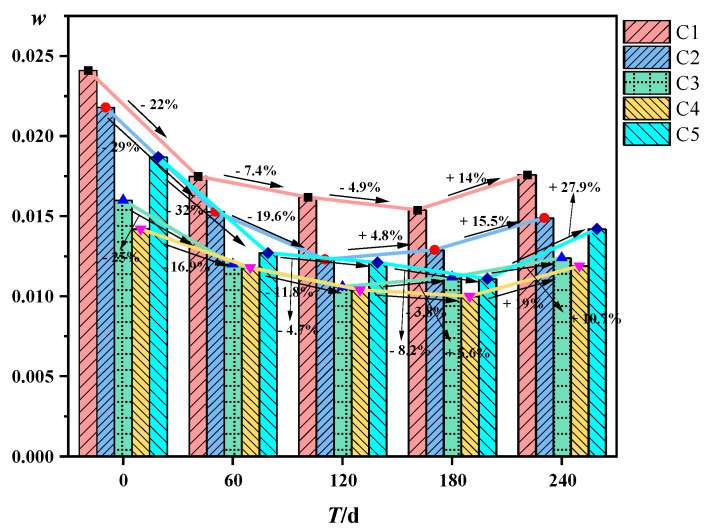
Concrete water absorption rate change.

As shown in Figure 7, the water absorption of the concrete test blocks shows a trend of decreasing and then increasing. The water absorption laterally reflects the porosity of the concrete structure, indicating that the pores in the structure first decrease and then increase. It is consistent with the conclusion obtained from the density of the concrete.

The cross-sectional comparison revealed that the compactness of C2 was better than that of C1, indicating that adding mineral admixtures can fill the pores in the concrete and make the concrete structure more compact than ordinary concrete. The fibers can inhibit the generation and development of cracks while improving the corrosion resistance of the concrete. The concrete densities in the C3, C4, and C5 mixes were consistently higher, and the water absorption rates were lower. Adding sodium methyl silicate and inorganic aluminum salt water repellent is beneficial to reduce internal defects, increase compactness, and improve concrete’s corrosion resistance.

### 4.2. Intensity

**Figure 8:**
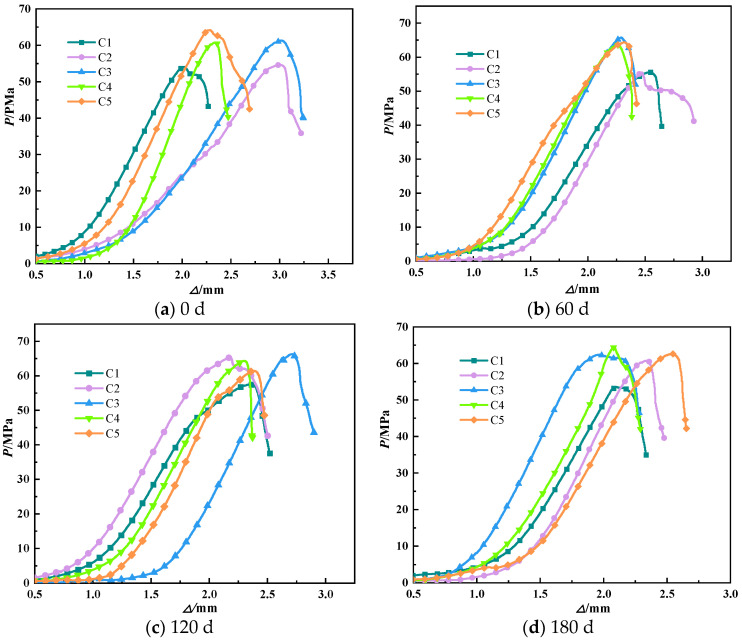

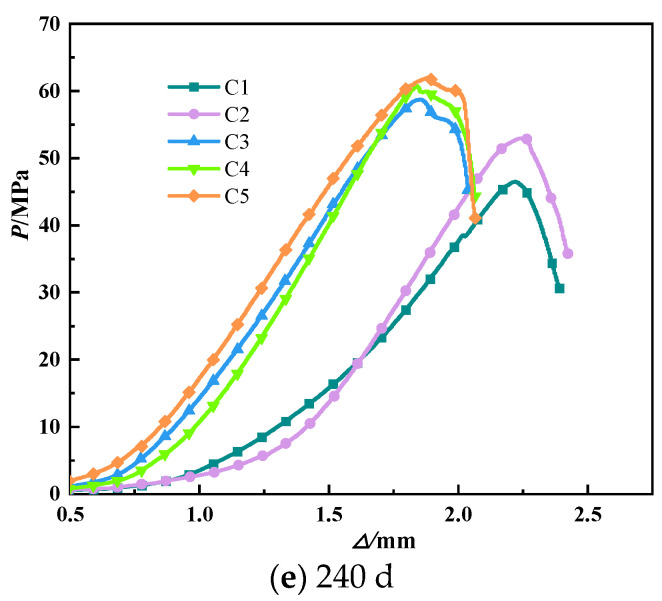
Load-displacement curve.

Tests were conducted to test the compressive strength of the concrete after corrosion, and the following results were obtained. The load-displacement curve of each mixing ratio test block is shown in Figure 8. Concrete compressive strength and ductility are significantly improved by adding basalt fiber, the mineral admixture and waterproofing agent. At 0 d, compared with C1, concrete’s maximum compressive bearing capacity with other mix ratios is greatly improved. It can be seen that mineral admixtures can fill pores, increase compactness and improve compressive ability. The displacement of concrete damage is extended, indicating that the ductility has been enhanced. Adding basalt fiber will improve the deformation capacity of concrete. With the increase of corrosion time, the bearing capacity decreases, and the biological acid in the organic aqueous solution corrodes the concrete to produce expansive substances, which reduces the strength; at the same time, the deformation capacity of concrete has fallen, from a displacement maximum of 3 mm down to about 2 mm.

The strength of concrete after corrosion is shown in Table 3. After 240 days of corrosion, the strength retention rate of ordinary concrete C1 is 85.86%, and the residual strength is 46.52 MPa. Compared with C1, the residual strength and strength retention rate of the other four groups is improved. Among them, C4 has the highest strength retention rate, 1.18 times that of C1; the residual strength is 60.79 MPa, 1.3 times that of C1. The strength retention rate and residual strength increased by 18% and 30%, respectively. After 240 days of corrosion, the strength has almost no attenuation. It can be seen that the inorganic aluminum salt waterproof agent has the best corrosion resistance effect on concrete. The strength retention rate of C3 and C5 doped with sodium methyl silicate is not as good as C4, but compared with C1, the strength retention rate is also increased by about 10%. Compared with C1, the strength retention rate of C2 increased significantly, indicating that basalt fiber and mineral admixtures can effectively resist sewage corrosion.

**Figure 9:**
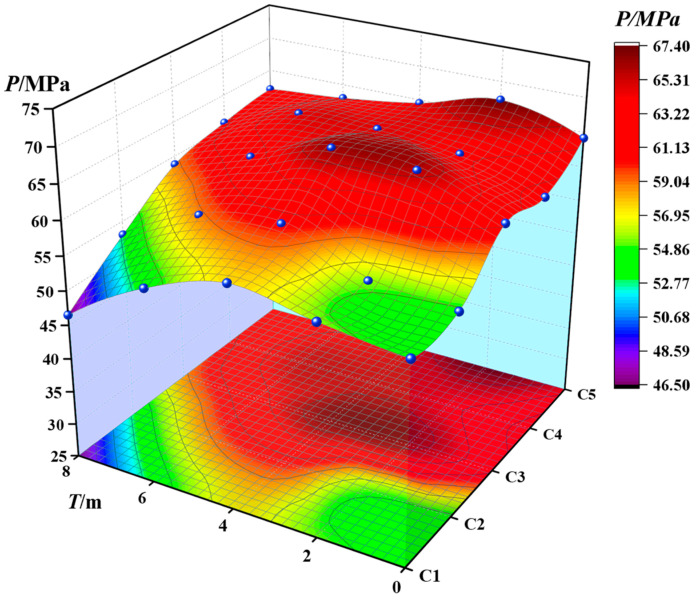
Spatial distribution of compressive strength.

Figure 9 shows the spatial variation of concrete strength. They corresponded to the data in Table 3. It can be seen that the strength of concrete increases first and then decreases with the increase of corrosion time, indicating that the internal porosity decreases first and then increases. Combined with the analysis of density change, it can be seen that the strength of concrete is related to density. The higher the density, the higher the strength.

To sum up, the plastic deformation ability is obviously improved by adding basalt fiber to concrete. Sodium methyl silicate and inorganic aluminum salt waterproofing agents can effectively improve concrete’s mechanical properties and corrosion resistance.

## 5. Discussion

The composition of the organic aqueous solution is complex, and concrete structures are easily eroded. The initial concrete surface is alkaline, and the solution contains sulfate ions, which are converted into H_2_S by anaerobic sulfate-reducing bacteria (SRB). H_2_S is further combined with oxygen to form H_2_SO_4_. These acidic compounds are neutralized with concrete so that the pH value of the concrete surface is gradually reduced. When the pH value is close to 4, the acidophilic SOB (ASOB) bacteria (such as *Acidithiobacillus ferrooxidans* and *Acidithiobacillus thiooxidans*) begin to multiply in large quantities, which will produce concentrated biological sulfuric acid, further acidify the environment, and then form biological sulfuric acid corrosion, which is the main reason for the deterioration of concrete performance.

**Figure 10:**
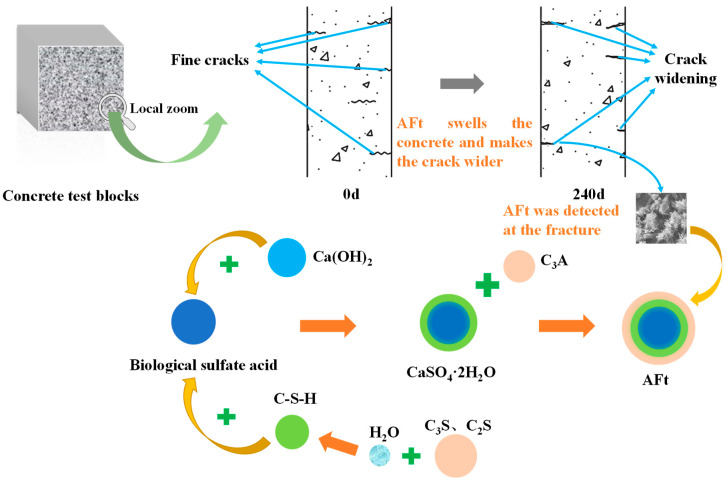
Concrete damage mode.

Concrete contains many calcium compounds (such as calcium hydroxide, C-S-H, calcium aluminate). Biosulfuric acid will initially react with Ca(OH)_2_ and later react with C-S-H gel to form gypsum, reducing the structural strength and increasing porosity. The gypsum will continue to react with hydrated calcium aluminate to create swelling ettringite, which will generate internal stress and eventually cause concrete cracking, as shown in Figure 10.

**Figure 11:**
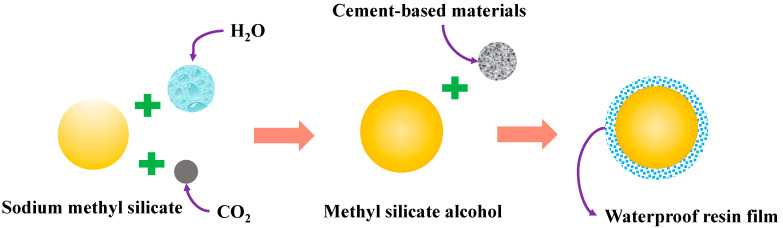
Sodium methyl silicate action model.

**Figure 12:**
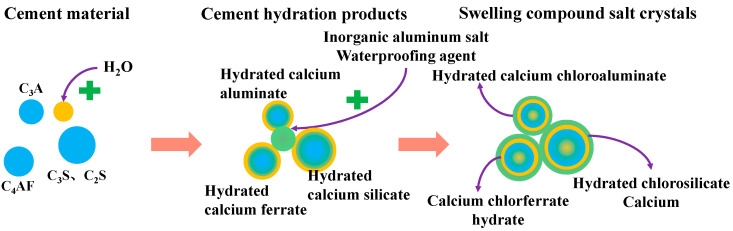
Inorganic aluminum salt waterproofing agent role model.

To lessen the deterioration of concrete performance, two kinds of waterproofing agents, sodium methyl silicate and inorganic aluminum salt, were added to prevent the penetration of harmful substances and protect concrete from corrosion. Sodium methyl silicate can form a layer of impermeable waterproof resin film on the surface of cement-based materials, as shown in Figure 11. Inorganic aluminum salt waterproof agent can generate an impermeable double salt inside the cement base, fill the pores, form a rigid waterproof layer, and effectively prevent harmful substances from entering, as shown in Figure 12. Finally, remote sensing technology can be used for real-time monitoring through the network to keep abreast of structural damage and reduce engineering losses [38,39,40].

Schematic diagram of the action model of sodium methyl silicate:

Inorganic aluminum salt waterproofing agent action model schematic diagram:

## 6. Conclusions

The following conclusions were mainly obtained by studying new basalt fiber concrete’s durability performance and corrosion mechanism under an organic water environment.

(1) After organic water solution corrosion, there are a lot of black algae deposits and crystallization on the concrete surface, and there is a sanding phenomenon. The addition of sodium methyl silicate and inorganic aluminum salt water repellent led to an improvement in the concrete surface corrosion. The effect of adding inorganic aluminum salt waterproofing agent is more prominent.
(2) Through microscopic analysis, it is found that there are many pores in ordinary concrete, and the pores of other mix ratios are significantly reduced. Among them, the test block with added inorganic aluminum salt waterproof agent is the most compact.
(3) The compressive strength analysis shows that the addition of inorganic aluminum salt waterproofing agent can increase the strength of the material by about 12% and the strength retention rate by 15%. The combination of basalt fiber and inorganic aluminum salt waterproofing agent has the best effect. It is recommended to be used in preference to normal concrete.
(4) The addition of an inorganic aluminum salt waterproofing agent significantly reduced corrosion and improved the durability of concrete materials in a sewage environment corrosion; the recommended dose of 6 kg/m^3^.
(5) This new highly durable concrete protection mechanism relies on basalt fiber and inorganic aluminum salt waterproofing agent, the addition of which causes filling of the internal pores, increased density of concrete materials and reduced rate of penetration of harmful substances. Sodium methyl silicate and cement-based materials react to form a specific waterproof layer, blocking ion penetration; however, the inorganic aluminum salt waterproofing agent protection effect is more prominent.

## Figures and Tables

**Table 1:** Concrete mix ratio (unit: kg/m^3^).

Types of Concrete	Cement	Sand	Crushed Stone	Water	Water Reducing Admixture	Basalt Fiber	Mineral Fines
C1	400	770	1155	160	3.6	0	0
C2	336	770	1155	160	3.6	2.5	80
C3	336	770	1155	160	3.6	2.5	80
C4	336	770	1155	160	3.6	2.5	80
C5	336	770	1155	160	3.6	2.5	80
**Types of concrete**	**Silica fume**	**Titanium dioxide**	**Alum**	**Quartz sand**	**Sodium methyl silicate**	**Inorganic aluminum salt**	
C1	0	0	0	0	0	0	
C2	20	0	1	5	0	0	
C3	20	0.5	1	5	1.2	0	
C4	20	0.5	1	5	0	6	
C5	20	0.5	1	5	1.2	6	

**Table 2:** High concentration organic water matching ratio (unit: g/20 kg).

Starch	Glucose	Peptone	Urea	Diammonium Phosphate	MgSO_4_	NaCl	Vitamin	Compound Fertilizer
368.64	201.216	55.68	24	12	180	180	6	20

Note: For every 20 kg of clear water, add 1 kg of sludge + 1 kg of algae-rich water as the biologic source.

**Table 3:** Compressive strength of concrete (unit: MPa).

Corrosion Time	C1	C2	C3	C4	C5
**0 d**	54.18	54.62	61.33	60.18	64.19
**60 d**	55.58	55.53	65.79	63.64	67.34
**120 d**	57.49	60.41	66.29	64.74	64.51
**180 d**	53.60	58.74	62.46	64.59	62.93
**240 d**	46.52	53.01	58.74	60.79	62.02

## Data Availability

Data are contained within the article.
